# Light harvesting in photosystem II

**DOI:** 10.1007/s11120-013-9824-3

**Published:** 2013-04-18

**Authors:** Herbert van Amerongen, Roberta Croce

**Affiliations:** 1 Laboratory of Biophysics, Wageningen University, P. O. Box 8128, 6700 ET Wageningen, The Netherlands; 2 Department of Physics and Astronomy, Faculty of Sciences, VU University Amsterdam, De Boelelaan 1081, 1081 HV Amsterdam, The Netherlands

**Keywords:** Excitation energy transfer, Picosecond fluorescence, Thylakoid membrane, Charge separation, State transitions

## Abstract

Water oxidation in photosynthesis takes place in photosystem II (PSII). This photosystem is built around a reaction center (RC) where sunlight-induced charge separation occurs. This RC consists of various polypeptides that bind only a few chromophores or pigments, next to several other cofactors. It can handle far more photons than the ones absorbed by its own pigments and therefore, additional excitations are provided by the surrounding light-harvesting complexes or antennae. The RC is located in the PSII core that also contains the inner light-harvesting complexes CP43 and CP47, harboring 13 and 16 chlorophyll pigments, respectively. The core is surrounded by outer light-harvesting complexes (Lhcs), together forming the so-called supercomplexes, at least in plants. These PSII supercomplexes are complemented by some “extra” Lhcs, but their exact location in the thylakoid membrane is unknown. The whole system consists of many subunits and appears to be modular, i.e., both its composition and organization depend on environmental conditions, especially on the quality and intensity of the light. In this review, we will provide a short overview of the relation between the structure and organization of pigment-protein complexes in PSII, ranging from individual complexes to entire membranes and experimental and theoretical results on excitation energy transfer and charge separation. It will become clear that time-resolved fluorescence data can provide invaluable information about the organization and functioning of thylakoid membranes. At the end, an overview will be given of unanswered questions that should be addressed in the near future.

## Introduction

The photosynthetic light reactions of green plants, algae, and cyanobacteria take place in photosystems I and II (PSI and PSII). Light-induced charge separation in the reaction center (RC) of PSII leads to the oxidation of water, the reduction of plastoquinone and the formation of a proton gradient across the thylakoid membrane in which PSI and PSII are embedded, which is crucial for the production of ATP. PSII and PSI work in series and together they also drive NADP^+^ to NADPH reduction with H_2_O as electron donor (Nelson and Yocum 2006). Light-induced charge separation in the RC of PSII starts from the primary donor P680 and an electron proceeds via a pheophytin onto plastoquinone *Q*_A_ and subsequently to plastoquinone *Q*_B_. The primary cation radical P680^+.^ has an *E*_m_ value of +1.25 V (Rappaport et al. 2009), far higher than the value of +0.80 for Chl in solution (Kobayashi et al. 2007) and this high value is ultimately responsible for the oxidation of water.

**Fig. 1:**
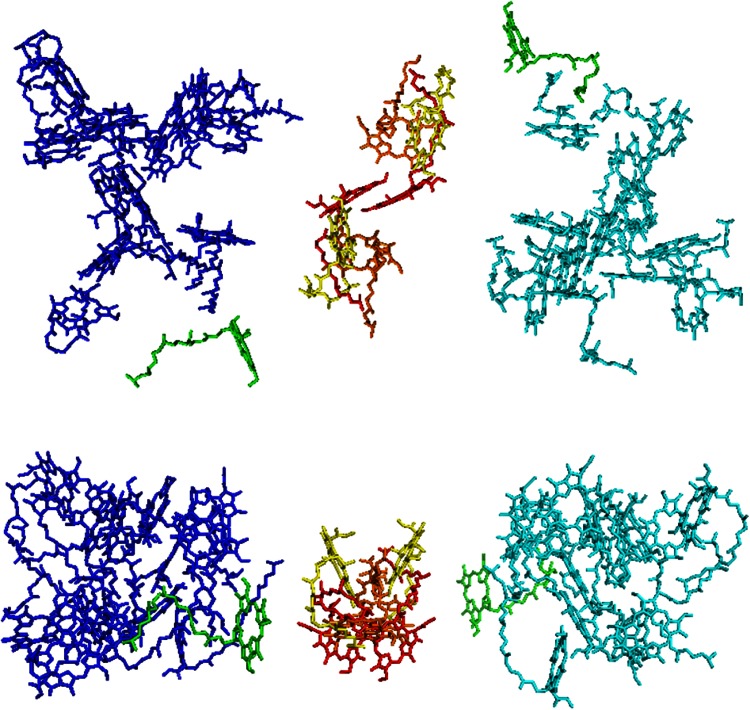
Chlorophyll organization in the core complex of PSII (Guskov et al. 2009). Chls P, *red*; Chls D1 and D2, *orange*; Chls z *green*; Pheos, *yellow*. The Chls of CP47 are in *blue* and those of CP43 in *cyan*. The phytol chains of the Chls are omitted for clarity. The upper figure shows a *top view* (from the stroma) and the lower figure provides a *side view*

The RC of PSII itself only contains six chlorophylls *a* (Chls *a*) and two pheophytins but it is always present in the so-called core complex that also contains the pigment-proteins CP43 and CP47, providing additional 13 and 16 Chls *a*, respectively, together with several β-carotene molecules (see (Umena et al. 2011) for the most recent PSII core structure). Both antenna complexes feed excitation energy into the RC. These antenna Chls are on the one hand at a “safe” distance from the RC pigments, which are highly oxidizing after charge separation (see Fig. 1), preventing direct pigment oxidation in the antenna, and on the other hand close enough to perform efficient excitation energy transfer (EET).

The core consists of ~20 different subunits, and the pigment/protein ratio is low which makes it a rather expensive piece of machinery. To increase the absorption cross-section further in a cost-effective way, additional light-harvesting complexes have appeared during evolution. Plants and green algae possess membrane-embedded antennae with a high pigment/protein ratio (~1:2 regarding the mass), also called outer light-harvesting complexes (Lhcs) and to a large extent they are organized in larger supercomplexes, together with the PSII cores. Cyanobacteria, that appeared earlier in evolution contain membrane-associated phycobilisomes (see e.g., (Neilson and Durnford 2010)) with a pigment-to-protein ratio that is substantially lower (~1:5) although still higher than for the core complex. For recent studies of EET in/from phycobilisomes in vitro and in vivo the reader is referred to Tian et al. (Tian et al. 2011, 2012). The present review will focus on light harvesting in plants.

The thylakoid membrane in plants is divided into grana, which are composed of stacks of membrane disks, and stroma lamellae, which connect the various grana in the choroplast (Mustardy and Garab 2003; Shimoni et al. 2005; Mustardy et al. 2008; Daum et al. 2010; Kouril et al. 2011). PSII is located in the grana (Andersson and Anderson 1980) whereas PSI is mainly present in the stroma lamellae (together with the ATP synthase). The thylakoid membrane is flexible and dynamic and able to respond to changes in environmental conditions by changing both composition and organization of the PSII supercomplexes (Anderson et al. 2008; Chuartzman et al. 2008; Goral et al. 2010). It has been shown that part of the grana membrane contains PSII arrays that consist of supercomplexes with different antenna sizes, but the abundance of the arrays seems to depend on the composition of PSII which for instance depends on the species analyzed and on the growth conditions (Boekema et al. 2000; Kouril et al.; Daum et al. 2010; Kirchhoff et al. 2007; Kouril et al. 2012; Kiss et al. 2008) (Kereiche et al. 2010; Kovacs et al. 2006; de Bianchi et al. 2008).

Only part of the PSII supercomplexes is embedded in these regular arrays, while another part is less organized. It is not exactly clear yet what the role of the arrays and the other parts is. But it is known that reorganizations in both arrays and other parts take place as a function of light quality and intensity (Wientjes et al. 2013; Kouril et al. 2012; Jahns and Holzwarth 2012; Betterle et al. 2009).

**Fig. 2:**
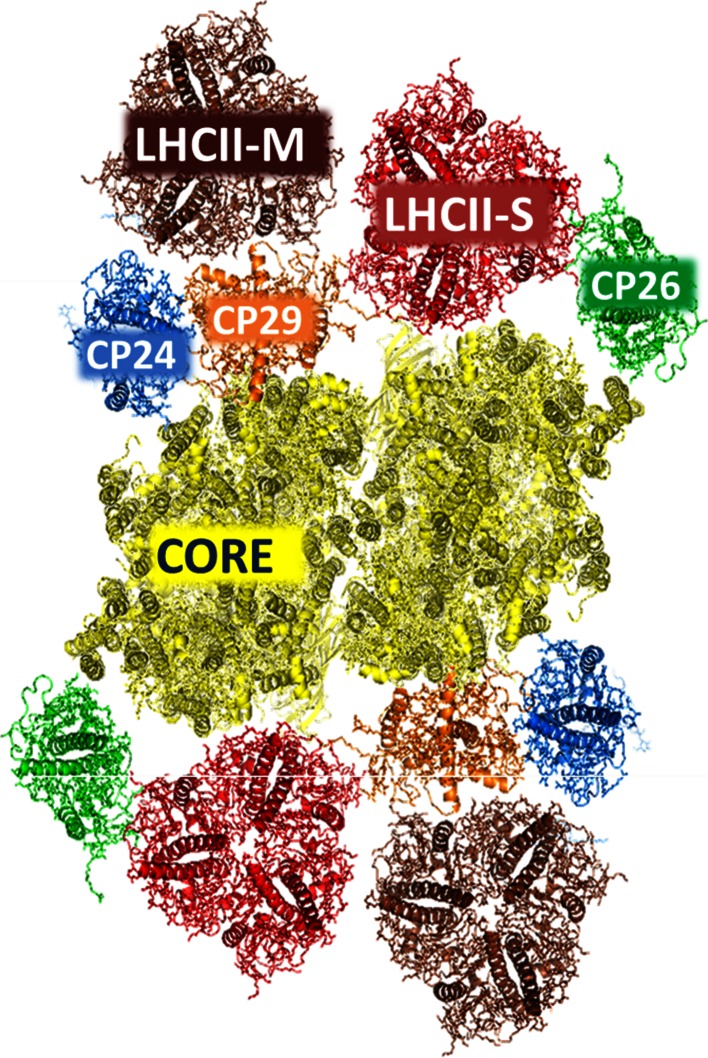
Model of the PSII supercomplex C2S2M2 from higher plants. *Top*-*view* for the stromal side on a C2S2M2 supercomplex from *A. thaliana*. Based on the projection map of C2S2M2 at 12 Å resolution, a model of the 3D structure of the supercomplex was reconstructed (Caffarri et al. 2009) using the crystal structures of PSII core (Guskov et al. 2009) and LHCII (Liu et al. 2004). For the minor antenna complexes, the structure of a monomer of LHCII was used while the pigment composition/occupancy was assigned based on the results of mutation analysis experiments on in vitro reconstituted complexes (Bassi et al. 1999; Remelli et al. 1999; Ballottari et al. 2009; Passarini et al. 2009)

In Fig. 2, a model of a plant PSII supercomplex is shown. It is composed of a PSII core together with the gene products of genes Lhcb1-6 in a well-defined arrangement. The largest supercomplexes contain a dimeric core, four LHCII (encoded by Lhcb1-3) trimers, two strongly bound (S) and two moderately strongly bound (M), and two monomeric copies each of CP29 (Lhcb4), CP26 (Lhcb5), and CP24 (Lhcb6). Supercomplexes of different sizes can be isolated (Caffarri et al. 2009), which is probably partly due to the solubilization process but it is also known that a sub-population of smaller supercomplexes is also observed in high light plants (see e.g., (Daum et al. 2010; Kouril et al. 2012)).

The Lhc complexes are densely packed with Chl *a* and *b* pigments and the xanthophylls lutein (Lut), violaxanthin (Vx), and neoxanthin (Nx) (with the exception of CP24 that does not contain Nx) which are responsible for light absorption and EET. Xanthophyll excitations (xanthophylls are carotenoids which contain oxygen) are rapidly transferred, typically within one ps to the Chls that are in Van der Waals contact with these carotenoids. Chls *b* transfer excitations to Chls *a*, which have lower excited-state energy, and on average only a small fraction of the excitations (~5 %) is located on Chl *b* molecules, due to Boltzmann equilibration in the excited state. Via rapid EET between mainly Chls *a* the excitations end up in the RC (see (Croce and van Amerongen 2011) for a review). Some of the Chl *a* singlet excitations are transformed into Chl *a* triplets, which can lead to the formation of destructive singlet oxygen molecules. Fortunately, most of these dangerous Chl triplets (>95 %) are scavenged by the carotenoids that are in Van der Waals contact with Chl *a* (Barzda et al. 1998; Lampoura et al. 2002; Mozzo et al. 2008a; Carbonera et al. 1992; van der Vos et al. 1991).

In this review, we will focus on the study of EET and CS in PSII, starting with the core, followed by outer antenna complexes and supercomplexes. A brief overview will then be given of results on thylakoid membranes, isolated from plants with varying antenna composition as a result of short- and long-term differences in light conditions. At the end, some unsolved problems will be presented together with suggestions for further research. We would also like to refer to other reviews from recent years for further information (Renger and Schlodder 2010; Vassiliev and Bruce 2008; Renger 2010; Van Amerongen et al. 2003; Minagawa and Takahashi 2004; Barber 2002; Muh et al. 2008; Renger and Renger 2008; Croce and van Amerongen 2011).

## The PSII core

**Fig. 3:**
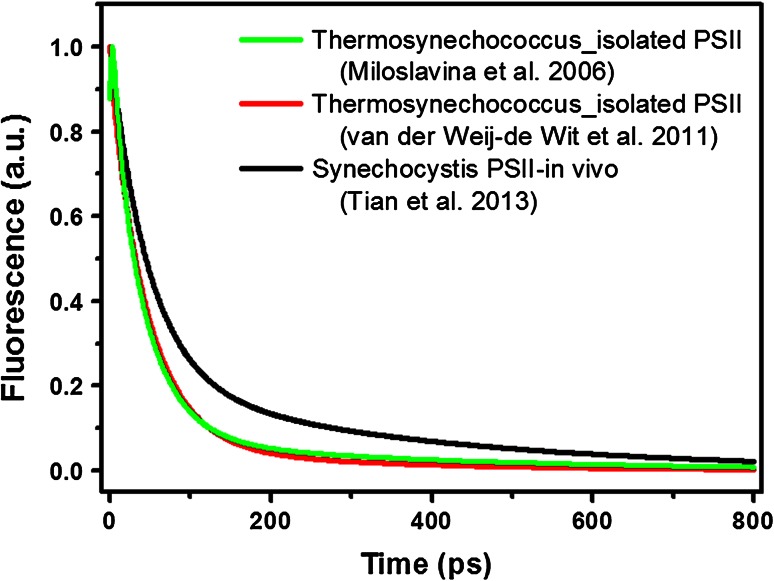
Picosecond kinetics of isolated PSII core complexes from *Thermosynechococcus*, reconstructed from (Miloslavina et al. 2006) (*black solid*) and (van der Weij-de Wit et al. 2011). The *decay curve* presented in (Miloslavina et al. 2006) was reconstructed based on the DAS shown in Fig. 7 of that work, and only *τ*1–*τ*5 are included in the calculation. The *decay curve* from (van der Weij-de Wit et al. 2011) was reconstructed based on the compartmental scheme shown in Fig. 6 in that article and the initial excitation fractions therein. Excitation wave lengths were 663 and 400 nm, respectively, but these differences are not expected to significantly influence the overall kinetics. The dotted line represents the fluorescence kinetics of PSII core in vivo for a *Synechocystis* mutant (excitation wavelength 400 nm) (Tian et al. 2013)

In Fig. 3, the reconstructed picosecond fluorescence kinetics of the PSII core from *Thermosynechococcus* from two different studies are shown (Miloslavina et al. 2006; van der Weij-de Wit et al. 2011) and the results are nearly identical. Accurate data fitting requires five or more exponentials but two direct observations stand out. Charge separation occurs with an average time constant *τ* below 100 ps, leading to the relatively fast disappearance of the (fluorescence) signal. Since this time constant is much shorter than the normal excited-state lifetime of chlorophyll (typically several ns), this guarantees a high quantum efficiency of charge separation (*ϕ*_CS_). The corresponding value is above 0.95, using the well-known relation *ϕ*_CS_ = 1 – *τ*/*τ*_Chl_ (Croce and van Amerongen 2011), where *τ*_Chl_ is the average lifetime of the excited Chl in PSII in the absence of charge separation. The exact value for this parameter is unknown but a recent study led to a value of ~2 ns (Belgio et al. 2012). The kinetics also shows a small contribution of a long-lived component which is usually ascribed to the fact that charge separation is partly reversible. The amplitude and lifetime of this component depend on the competition between secondary charge separation in the RC (forward electron transfer from the primary electron acceptor) and back transfer of the electron from primary acceptor to primary donor.

Although the kinetics in both studies is rather similar, the models that were used for the fitting differ considerably. It should be noted that the overall (average) trapping time *τ* of excitations can in good approximation be considered as the sum of two terms: *τ* = *τ*_mig_ + *τ*_trap_ (Van Amerongen et al. 2000; Broess et al. 2006). In a trap-limited model, the equilibration time (also called migration time *τ*_mig_) of excitations over the photosystem is assumed to be much shorter than the overall trapping time, i.e., it can largely be neglected and thus *τ* = *τ*_trap_. The best-known trap-limited model is the so-called exciton/radical pair equilibrium model (ERPE model) (van Grondelle 1985; Schatz et al. 1988, 1987), and it has widely been used to interpret all kinds of variations in fluorescence in photosynthesis. Besides primary charge separation, it also includes charge recombination and secondary charge separation (see above). In (Miloslavina et al. 2006), the data were fitted to a kind of trap-limited model and it was thus assumed that excitation equilibration in the core occurs on a time scale much faster than the overall trapping time. In good approximation *τ*_trap_ is equal to the intrinsic charge separation time of the primary donor *τ*_iCS_, divided by the probability that the excitation is actually located on the primary donor. For *N* isoenergetic pigments, including the primary donor, *τ*_trap_ = *N* *τ*_iCS_ (when charge recombination is ignored). Taking for instance values of *τ*_trap_ = 60 ps and *N* = 35, one finds that *τ*_iCS_ = 1.7 ps.

However, the distances between the pigments in these complexes and the ones in the RC (Fig. 1) are so large that it was concluded in (van der Weij-de Wit et al. 2011) that the transfer time of excitations to the trap and therefore the contribution of *τ*_mig_ cannot be ignored. This means that the value of *τ*_trap_ should be smaller and concomitantly the same should be true for *τ*_iCS_, which also comes out of the fitting (van der Weij-de Wit et al. 2011). Very recently, the picosecond fluorescence kinetics was obtained for the PSII core in vivo, by comparing the results of different mutants of *Synechocystis* PCC *6803* mutants (Tian et al. 2013). It turned out that the PSII core of this organism in vivo was somewhat slower than the one of *Thermosynechococcus* in vitro but again, the kinetics could be satisfactorily fitted with both a trap-limited and a migration-limited model. It is clear that comparing different fitting models cannot favor one trapping model above the other.

In a recent theoretical treatment Raszewski and Renger (Raszewski and Renger 2008) concluded that the trapping should be migration-limited: Transfer from CP43/CP47 occurs with time constants of 40–50 ps. The main reason for the slow transfer is the large distance between the pigments in the core antenna and those in the RC. As was mentioned above, this large distance is probably needed to avoid oxidation of the antenna pigments. The consequence of this slow EET is that the primary charge transfer time should be extremely fast, i.e., around 300 fs, accompanied by a very large initial drop in free energy to explain the overall time-resolved results. It should be noted that at least in isolated RC complexes such a fast charge separation time was not observed (Groot et al. 2005; Germano et al. 2004; van Mourik et al. 2004; Holzwarth et al. 2006; Prokhorenko and Holzwarth 2000; Andrizhiyevskaya et al. 2004; Wasielewski et al. 1990; Durrant et al. 1992; Pawlowicz et al. 2008) and one might wonder whether this is realistic. On the other hand, it is possible that isolated RC complexes are “slower” than the ones in vivo (see also below).

It is worthwhile to mention that the average lifetimes of core preparations from cyanobacteria are in general far shorter than for cores from plants (Raszewski and Renger 2008). Although this may be due to differences in the intrinsic properties of the cores, it is most likely related to problems associated with the isolation of core preparations from plants.

At the moment, there are still several unsolved issues with respect to PSII core kinetics. Both trap- and migration-limited models seem to have some intrinsic problem and maybe we should consider the possibility of coherent EET into the RC (Collini and Scholes 2009). Moreover, we do not know to which extent observed differences between in vivo and in vitro results are due to differences in biological species or to the fact that the thylakoid membrane might influence the primary EET and CT steps. Why do cores isolated from plants show slower kinetics than those of cyanobacteria? We have not mentioned any studies on the kinetics of isolated RCs but as was discussed in (Broess et al. 2006), they appear to be substantially slower than when embedded in larger systems. Therefore, it seems that the study of isolated complexes at the moment can only contribute to basic knowledge about charge separation mechanisms and pathways in PSII but they do not give realistic time constants.

## Outer antenna complexes

**Fig. 4:**
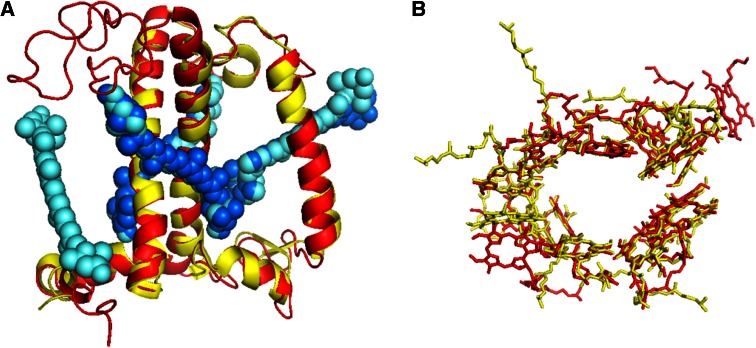
Overlap of the structural models of LHCII (Liu et al. 2004) and CP29 ((Pan et al. 2011)). **a** *Side view* (from within the membrane) on the protein backbone of LHCII (*red*) and CP29 (*yellow*) and the xanthophylls of LHCII (*light blue*) and CP29 (*dark blue*). Main differences are the lack of the *N*-terminal part of CP29 which apparently was cleaved off during crystallization and the lack of VX in CP29. For the rest, both proteins show very similar structures. **b** *Top view* showing that the Chl organization in LHCII (*red*) and CP29 (*yellow*) is rather similar although not identical

The antenna complexes of PSII from higher plants are composed of members of the Lhc multigenic family. The structure of a monomeric subunit of trimeric LHCII (Liu et al. 2004; Standfuss et al. 2005) is given in Fig 4. Each monomer coordinates eight Chls *a*, six Chls *b* and four xanthophylls (one Nx, two Lut’s and one Vx). The two Lut’s are located at sites L1 and L2 in the center of the molecule while Nx and Vx are located at the periphery in sites N1 and V1, respectively (Croce et al. 1999; Caffarri et al. 2001; Ruban and Horton 1999). The average distance between the Chls is around 10 Å, which leads to excitonic interactions between the pigments, resulting in fast energy transfer within the complex.

Based on sequence similarity, all members of the Lhc family are thought to have a similar structural arrangement (Green and Khlbrandt 1995) and most of the amino acids that bind Chl in LHCII are conserved in all family members (Bassi et al. 1997). This is now confirmed for CP29 and Lhca1-4 based on the comparison of the structures (Pan et al. 2011; Amunts et al. 2010). The structures of CP29 and monomeric LHCII are shown in Fig. 4. Nevertheless, individual complexes show different biochemical and spectroscopic properties (see e.g., (Pascal et al. 1999)), mainly due to the fact that the pigment composition is not identical (Sandona et al. 1998). Mutations of the putative Chl-binding residues followed by in vitro reconstitution (Plumley and Schmidt 1987) has allowed the characterization of the chromophores in most binding sites (Bassi et al. 1999; Remelli et al. 1999; Yang et al. 1999; Rogl and Kuhlbrandt 1999; Ballottari et al. 2009; Passarini et al. 2009). The four Chl-binding sites in the center (602, 603, 610 and 612) accommodate Chls *a* in all antenna complexes. Chls 602–603 absorb around 675 nm and Chls 610–612 absorb around 680 nm, representing the lowest energy state(s) of the system (Remelli et al. 1999; Rogl and Kuhlbrandt 1999). The domain including helix C mainly coordinates Chls *b* (Remelli et al. 1999; Peterman et al. 1996). In all complexes, site L1 contains a Lut while L2 accommodates Lut in LHCII and CP26 but Vx in CP29 and CP24. Nx is present in the N1 site of all complexes apart from CP24 (Caffarri et al. 2007).

By combining the results of a large number of different studies on LHCII in the nineties (Visser et al. 1996; Savikhin et al. 1994a; Peterman et al. 1997; Croce et al. 2001; Connelly et al. 1997), it was concluded that (sub)picosecond EET leads to ps spectral equilibration and excitations become mainly localized on the peripheral Chl *a* pigments on the stromal part of the protein i.e., Chls 610–612 (Van Amerongen and van Grondelle 2001). From there, they can be transferred to neighboring complexes in the thylakoid membrane. Spatial equilibration within the trimers occurs on a slower time scale (tens of ps) as was concluded from several other studies (Savikhin et al. 1994b; Barzda et al. 2001; van Oort et al. 2007; Kwa et al. 1992; Novoderezhkin and van Grondelle 2010).

The results of the various time-resolved and steady-state spectroscopic studies were later modeled with the use of Redfield theory (Novoderezhkin et al. 2004, 2005) and led to a theoretical description of the data largely consistent with the crystal structure (Liu et al. 2004; Standfuss et al. 2005), demonstrating that within a few ps, the excitations are mainly localized on Chls 610–612.

More recent studies using 2-D electronic spectroscopy (Calhoun et al. 2009) are at least qualitatively in agreement with the modeling results of Novoderezhkin et al. (Novoderezhkin et al. 2005; Novoderezhkin and van Grondelle 2010) although it is not known whether the new models also lead to a correct description of for instance the linear-dichroism (LD) (Van Amerongen et al. 1994) and circular-dichroism (CD) spectra (Georgakopoulou et al. 2007). It is worth to point that in a very recent study, Müh and Renger were able to obtain rather satisfactory fits of all steady-state spectra of LHCII, demonstrating that not all site energies agree with those obtained before and that also the absolute LD spectra do not perfectly agree with the crystal structure (Muh and Renger 2012). Therefore, it seems that there is room for an additional round of improving both the structural model of LHCII and the understanding of its steady-state and time-resolved spectroscopic properties. At this point, it is also worth to mention that Zucchelli et al. (Zucchelli et al. 2012) recently calculated LHCII absorption spectra and obtained substantial variation for the monomeric subunits of three different trimers taken from the crystal structure (Liu et al. 2004; Standfuss et al. 2005), due to variations in the conformation of the Chl macrocycle and variations in the excitonic coupling strength between different Chls. Finally, it is worth mentioning that the (sub)ps transient absorption kinetics of the three gene products forming LHCII, Lhcb1, Lhcb2, and Lhcb3, are identical (Palacios et al. 2006).

EET in the minor antenna complexes (Cinque et al. 2000; Gradinaru et al. 1998, 2000; Salverda et al. 2003; Croce et al. 2003a, b; Marin et al. 2010, 2011) seems to occur along similar pathways as in LHCII. Also in these complexes equilibration occurs within a few ps, leading to excitation population mainly on Chls 610–612, the lowest energy pigments located on the stromal side at the periphery of the complex (Mozzo et al. 2008b).

## PSII supercomplexes

Obtaining homogeneous preparations of PSII supercomplexes is difficult because they disassemble quite easily (Wientjes et al. 2009; Caffarri et al. 2001). The largest supercomplex purified so far is C2S2M2 (Fig. 2) (Caffarri et al. 2009) and it is the most abundant complex in thylakoid membranes of *Arabidopsis thaliana* (Dekker and Boekema 2005; Kouril et al. 2012). The LHCII trimers differ somewhat in composition. The S trimer is composed of the products of the Lhcb1 and Lhcb2 genes and the M trimer in addition also contains the product of the Lhcb3 gene (Hankamer et al. 1997). Ordered arrays of C2S2, C2S2M, and C2S2M2 have been observed in membranes of different plants (Boekema et al. 2000; Daum et al. 2010; Yakushevska et al. 2001; Kouril et al. 2011). Smaller supercomplexes have also been purified but they are probably partly disassembled (Caffarri et al. 2009). Based on a projection map of the C2S2M2 supercomplex at 12 Å resolution (Caffarri et al. 2009) and the crystal structures of core and LHCII, a 3D supercomplex structure has been reconstructed (Fig. 2). Such a model can be used to visualize possible EET pathways (Croce and van Amerongen 2011).

Picosecond fluorescence measurements have been performed on four different PSII supercomplex preparations from *A. thaliana* (Caffarri et al. 2011). The smallest complex (C2S) contains a dimeric PSII core plus CP26, CP29 and one LHCII trimer. The largest complex (C2S2M2) corresponds to the structure in Fig. 2. The average fluorescence lifetime becomes longer upon increasing the antenna size from 109 ps for the dimeric core complex (~70 Chl *a* molecules) to 158 ps for C2S2M2 (~210 Chl *a* molecules), using a detergent concentration of 0.01 % α-DM. In 0.001 % α-DM the lifetimes decrease on average by around 20 ps. Plotting the average lifetimes versus the number of Chls *a* for the four supercomplex preparations and the core, shows that all values lie more or less on a straight line which evidently is not going through the origin as one might expect (Van Amerongen et al. 2003) indicating that the energy transfer and/or charge separation rates are reduced in complexes with decreased size, strongly suggesting that the antenna system is important for plant PSII integrity and functionality, which is also supported by biochemical results (Caffarri et al. 2011).

The kinetics were also simulated using coarse-grained modeling and the obtained parameters were used to illustrate various aspects of PSII functioning (Caffarri et al. 2011). It was for instance calculated that for the largest supercomplex the efficiency of charge separation is 89 %. In the presence of one open and one closed RC, the photochemical efficiency reduces to 78 %, which is much larger than the value of 45 % calculated when the cores are not connected into dimers. This demonstrates that a dimeric conformation increases the light-harvesting capacity by more than 70 % in the presence of one closed RC. This is an important property for PSII because of its slow turnover and it also suggests that the arrays of PSII that are observed in electron-microscopy measurements are advantageous when a substantial fraction of the RC’s is closed.

In fact, the advantage of PSII units being connected to each other was already discussed many decades ago and it was experimentally determined that indeed many “photosynthetic units” (PSU’s) are connected to each other (see e.g., (Clayton 1981)). Two popular models from those days were the puddle model, in which PSU’s were not connected and the lake model, in which basically all PSU’s were connected. Whereas for purple bacteria, the lake model is applicable, it was found that for plants, the situation was somewhere in between these extreme models (see e.g., also (Clayton 1981)), which is in agreement with the organization observed with electron-microscopy (see above).

## Energy transfer and charge separation in PSII membranes

Grana membranes can be purified (the so-called BBY particles) that contain practically only PSII complexes (Berthold et al. 1981; Dunahay et al. 1984; Albertsson et al. 1981), although it is not completely understood how PSII is organized in these membranes. It had been suggested that C2S2 represents the supercomplex in high light, while C2S2M2 is the result of low-light growth (Daum et al. 2010). However, it was recently demonstrated that also in high light, C2S2M2 is still the main supercomplex in Arabidopsis (Kouril et al. 2012). In high light, the amount of LHCII trimers is lower than in low light, although in all cases the stoichiometry LHCII/core is higher than two (it is often between three and four) (Bailey et al. 2001; Anderson and Andersson 1988; Kouril et al. 2012), meaning that not all LHCII trimers are present in the supercomplexes but that there are also “extra” trimers. The location of these “extra” LHCII trimers, however, is still unknown and some of them might be located in the LHCII-only domains that were proposed by Boekema et al. (Boekema et al. 2000) although it should be emphasized that most of the “extra trimers” should be connected to PSII which is not necessarily the case for these LHCII-only domains. Part of these extra trimers attaches to PSI in the presence of light but detaches again in the dark and also during light-stress conditions (Wientjes et al. 2013). Recently, it was also found that in Arabidopsis plants, the amount of M trimers is decreasing when the grow-light intensity is increased from 100 to 800 μmol photons m^−2^ s^−1^, whereas the amount of “extra” trimers remains the same. Decreasing on the other hand the intensity to 20 μmol photons m^−2^ s^−1^, leads to an increase in the amount of “extra” trimers, whereas the amount of M trimers now remains unaltered (Kouril et al. 2012). For nearly all time-resolved studies in the literature, detailed information about the antenna composition is lacking.

In the past, various studies have been performed on BBY preparations (Berthold et al. 1981). The kinetics of these membranes were for instance described by a single lifetime of 210 ps (Schilstra et al. 1999) or with a major lifetime of 140 ps and a minor lifetime of 330 ps (Van Mieghem et al. 1992). More recently, two studies were done that showed average lifetimes in the order of 150–160 ps (Broess et al. 2006, 2008) and the results were interpreted with a coarse-grained model that uses the C2S2M2 structure as a basis. Like in the ERPE model, it was assumed that primary charge separation (with rate *k*_CS_ or inverse rate/transfer time *τ*_CS_) is reversible (first charge-separated state is Δ*G* lower in energy than the state in which the RC is excited in the *Q*_y_ state). Secondary charge separation (with rate *k*_RP_ or inverse rate/transfer time *τ*_RP_) was supposed to be irreversible. EET was modeled by assuming hopping to occur between neighboring (monomeric) complexes with a rate called *k*_*h*_ (or inverse rate/hopping time *τ*_*h*_) that was assumed to be the same for all hopping steps, whereas each rate was scaled with the number of pigments per complex. The basic difference with the earlier ERPE model is the fact that the supercomplex is used as a structural model to include EET steps and the fact that the hopping rate is not assumed to be infinitely fast. Using this model it was shown that different combinations of *τ*_CS_ and *τ*_*H*_ can describe the data nearly equally well (Broess et al. 2006), reminiscent of the data fitting results for core samples. Although it was not possible to extract more details about the charge transfer kinetics in the RC, it was possible to conclude that the BBY data could not be explained with published parameters for charge separation as obtained from time-resolved studies on cores by for instance Vasilliev et al. (Vassiliev et al. 2002) and Miloslavina et al. (Miloslavina et al. 2006) and other studies. Good resemblance could only be obtained when both the rate of charge separation and the drop in free energy upon charge separation were increased. It was also argued that previously published results on isolated PSII RC (Andrizhiyevskaya et al. 2004; Groot et al. 2005) were not in accordance with the BBY results. Again, this seems to indicate that the antenna system is important for plant PSII integrity and functionality.

In a follow-up study (Broess et al. 2008), time-resolved fluorescence measurements were performed on PSII membranes using two different excitation wavelengths, 420 and 483 nm. In this way, the relative number of excitations in core and outer antenna was varied, and the migration time from outer antenna to core was estimated to be 20–25 ps, much faster than one might expect based on earlier results on random aggregates of LHCII (Barzda et al. 2001). Therefore, it seems that the organization of the light-harvesting complexes in the supercomplexes/PSII membranes has been optimized in such a way that efficient EET takes place. However, at the moment detailed EET calculations are still lacking.

## Energy transfer and charge separation in PSII in the thylakoid membrane

Isolated thylakoid membranes contain all complexes participating in the light reactions of photosynthesis but the large heterogeneity of the system and the presence of different complexes strongly complicate the interpretation of the time-resolved data. In general, the kinetics of thylakoids with open RCs are multi-exponential with lifetimes ranging from tens of picoseconds to values between 300 and 600 ps, and the average lifetime typically ranges from 300 to 400 ps (Engelmann et al. 2005; Leibl et al. 1989; Roelofs et al. 1992; Vasil’ev et al. 1998). However, interpretation of the individual lifetimes has remained ambiguous (for an overview see also (van Grondelle et al. 1994; Van Amerongen et al. 2003)). Recently, thylakoid membranes from *A. thaliana* with 4 LHCII trimers per RC were studied using various detection wavelengths to discriminate between PSI and PSII kinetics. Making use of two excitation wavelengths, it was possible to estimate the migration time from PSII outer antenna to core (van Oort et al. 2010). The fluorescence decay could be fitted very well with three lifetimes, in this particular case being 73, 251, and 531 ps (plus a very small contribution of a ns component) at all wavelengths with varying amplitudes. Shorter lifetimes mainly reflect spectral equilibration within individual complexes (see above) and are of less interest for the entire membrane. The three main lifetimes are sufficient to describe the data although they do not directly correspond to well-defined physical processes, and they are the result of different processes and heterogeneity in the membrane. Note that these lifetimes usually differ for different preparations, depending on for instance growth-light conditions and the state of the membrane (light- or dark-adapted, state 1 or state 2, in the presence or absence of nonphotochemical quenching (NPQ) and with open or closed RCs). The shortest of these three lifetimes (fitted with 73 ps in this case) is partly due to PSI whereas the other two are almost exclusively due to PSII as can be concluded from the shapes of the decay-associated spectra (van Oort et al. 2010). In this particular case, the thylakoids were dark-adapted and therefore in state 1 (no LHCII attached to PSI) with open RCs and in the absence of NPQ. It was estimated that *τ*_trap_ = 180 ps and *τ*_mig_ = 150 ps. This migration time is a factor of 4–5 longer than for the PSII membranes above, which contained 2.4–2.5 trimers per RC. Therefore, it is clear that the extra trimers are connected less well to the RCs. These results indicate that at the level of the thylakoid membrane trap-limited models are certainly not valid. At this point, it is also worth mentioning that different supercomplexes are functionally connected to each other and the domain size (how far does/can an excitation travel?) was estimated to be 12–24 LHCII trimers by Lambrev et al.(Lambrev et al. 2011).

In (Wientjes et al. 2013) it was studied for *A. thaliana* how the time-resolved fluorescence kinetics depends on the distribution of LHCII over PSI and PSII. In most light conditions some LHCII is attached to PSI (at most one LHCII trimer per PSI, on average around half a trimer). PSI and PSI-LHCII contribute only to the fastest (87 ps in this study) component to which also PSII contributes. Lifetimes of 0.26 and 0.54 ns are due to PSII and are very similar to the lifetimes reported above, namely 0.25, and 0.53 ns (van Oort et al. 2010) The longest lifetime is only observed in the presence of “extra” LHCII and is for instance not found for supercomplexes or PSII membranes with only 2.5 LHCII trimers per RC (see above). Upon relocation of LHCII from PSII to PSI the relative amplitude of the 87 ps component increases at the expense of the 0.26 and 0.54 ns components. This is explained by a decreased contribution of the “extra” LHCIIs to the “slow” PSII fluorescence decay, and an increased contribution to the ~87 ps component by PSI-LHCII, thereby shortening the average fluorescence lifetime of the thylakoids.

## Where to go?

At the level of the individual pigment-protein complexes the functioning of the outer light-harvesting complexes of PSII seems to be relatively well understood ("Outer antenna complexes" section). When it comes to the PSII core, there is more uncertainty ("The PSII core" section, ). Different labs are able to obtain very similar experimental results on the same samples but there is strong disagreement about the interpretation. Moreover, there seem to be differences between the “performance” of core complexes in vitro and in vivo and striking differences exist between core preparations from plants and cyanobacteria, although it is generally assumed that these cores are very similar. However, the cores in plants are surrounded by outer light-harvesting complexes, which is not the case in cyanobacteria. It is clear from the work on PSII supercomplexes that the intrinsic performance of the core of PSII is improving when the supercomplexes increase in size ("PSII supercomplexes" section). For PSII membranes, the performance seems to be even better and one might wonder to which extent the stacking of the membranes in the grana contributes to the overall performance. It is for instance still unknown how efficient EET between different membrane layers is: At the moment, the existing models mainly include EET within individual layers. It should, however, be noted that studies of Kirchhoff et al. (Kirchhoff et al. 2004) and Lambrev et al. (Lambrev et al. 2011) suggested that unstacking of the different membrane layers has no noticeable effect on excitation energy transfer, thereby implying that transfer between membrane layers is not very important. The modeling is not very sophisticated yet, which is partly due to the fact that also the structural models are not very accurate and good models should somehow also incorporate the structural variability of the membranes (in addition to heterogeneity): membranes are dynamic systems.

In thylakoid membranes where the average number of LHCII trimers can go up to four, depending on light conditions, the migration time is considerably slower, demonstrating that on the thylakoid level the charge separation process is definitely not trap-limited. It is still not known where the extra antenna complexes are located, but it is also not known to which extent they are disconnected and to which extent these complexes are quenched. There is a clear need for further studies on the grana organization and composition in different (light) conditions to enable more detailed modeling studies.

Finally, it will be very important to perform time-resolved studies in vivo, preferably at the single chloroplast level, using microscopic techniques. Only then will it be possible to see the “real” photosynthesis in action; after all, it is a very flexible and dynamic process and the chloroplast is continuously adapting to changing conditions.
